# Adjuvanting Allergen Extracts for Sublingual Immunotherapy: Calcitriol Downregulates CXCL8 Production in Primary Sublingual Epithelial Cells

**DOI:** 10.3389/fimmu.2020.01033

**Published:** 2020-06-09

**Authors:** Michael P. Pelst, Clara Höbart, Charlotte Wallaeys, Hilde De Rooster, Yannick Gansemans, Filip Van Nieuwerburgh, Bert Devriendt, Eric Cox

**Affiliations:** ^1^Laboratory of Immunology, Department of Virology, Parasitology and Immunology, Faculty of Veterinary Medicine, Ghent University, Merelbeke, Belgium; ^2^Center of Physiology and Pharmacology, Medical University of Vienna, Vienna, Austria; ^3^VIB Center for Inflammation Research, Ghent, Belgium; ^4^Department of Biomedical Molecular Biology, Ghent University, Ghent, Belgium; ^5^Small Animal Department, Faculty of Veterinary Medicine, Ghent University, Merelbeke, Belgium; ^6^Laboratory for Pharmaceutical Biotechnology, Faculty of Pharmaceutical Sciences, Ghent University, Ghent, Belgium

**Keywords:** sublingual, epithelium, *Dermatophagoides farinae*, Toll-like receptor, calcitriol, CXCL8, dog, sublingual immunotherapy

## Abstract

Application of allergens onto the sublingual epithelium is used to desensitize allergic individuals, a treatment known as sublingual immunotherapy. However, the response of sublingual epithelial cells to house dust mite allergen and potential tolerance-promoting adjuvants such as Toll-like receptor (TLR) ligands and calcitriol has not been investigated. In order to study this, primary sublingual epithelial cells were isolated from dogs and cultured *in vitro*. After 24-h incubation with a *Dermatophagoides farinae* extract, a *Dermatophagoides pteronyssinus* extract, TLR2 ligands (FSL-1, heat-killed Listeria monocytogenes, Pam3CSK4), a TLR3 ligand (poly I:C), a TLR4 ligand [lipopolysaccharide (LPS)], and calcitriol (1,25-dihydroxyvitamin D_3_), viability of the cells was analyzed using an MTT test, and their secretion of interleukin 6 (IL-6), IL-10, CXCL8, and transforming growth factor β1 (TGF-β1) was measured by enzyme-linked immunosorbent assay. Additionally, to evaluate its potential effect as an adjuvant, sublingual epithelial cells were incubated with calcitriol in combination with a *D. farinae* extract followed by measurement of CXCL8 secretion. Furthermore, the effect of *D. farinae* and calcitriol on the transcriptome was assessed by RNA sequencing. The viability of the sublingual epithelial cells was significantly decreased by poly I:C, but not by the other stimuli. CXCL8 secretion was significantly increased by *D. farinae* extract and all TLR ligands apart from LPS. Calcitriol significantly decreased CXCL8 secretion, and coadministration with *D. farinae* extract reduced CXCL8 concentrations to levels seen in unstimulated sublingual epithelial cells. Although detectable, TGF-β1 secretion could not be modulated by any of the stimuli. Interleukin 6 and IL-10 could not be detected at the protein or at the mRNA level. It can be concluded that a *D. farinae* extract and TLR ligands augment the secretion of the proinflammatory chemokine CXCL8, which might interfere with sublingual desensitization. On the other hand, CXCL8 secretion was reduced by coapplication of calcitriol and a *D. farinae* extract. Calcitriol therefore seems to be a suitable candidate to be used as adjuvant during sublingual immunotherapy.

## Introduction

Allergen-specific immunotherapy (ASIT) is currently the only therapy able to alter the immunopathological reactions during allergies, teaching the immune system to tolerate the specific allergen (1). Sublingual immunotherapy (SLIT) is a form of ASIT during which allergens are administered under the tongue onto the sublingual epithelium (2, 3). Epithelial cells are known to be immunologically responsive and to influence the behavior of surrounding immune cells. For instance in allergic disease, epithelial cells can secrete interleukin 25 (IL-25), IL-33, and thymic stromal lymphopoietin (TSLP), which promote proallergenic responses (4, 5). House dust mites are one of the most important allergen sources, causing respiratory allergies in humans (6) and atopic dermatitis in dogs (7). House dust mite allergens are known to interact with Toll-like receptors (TLRs) and protease-activated receptors expressed by epithelial cells. This induces proinflammatory and proallergenic mediator production in human airway epithelial and epidermal cell cultures (8–11) and contributes to disease development in mouse asthma models (12–14). However, whether sublingual epithelial cells are responsive to house dust mite allergen extracts has not been assessed.

During SLIT, the use of tolerance-promoting adjuvants has been suggested to improve the number of successfully treated patients and to expedite the clinical onset of allergen-specific desensitization (15). Toll-like receptor ligands have been proposed as such adjuvants. In a mouse asthma model, sublingual coadministration of an antigen and the synthetic TLR2 ligand Pam3CSK4 decreased airway hyperresponsiveness and reduced antigen-specific T helper 2 (T_H_2) responses in the draining cervical lymph nodes (16). In addition, *ex vivo* exposure of human peripheral blood mononuclear cells to TLR2 ligands reduced their IL-5, IL-13, and immunoglobulin E (IgE) production (17, 18), suggesting a suppressing effect of TLR2 ligands on T_H_2-mediated responses. Apart from TLR2, TLR4 ligands were reported to have a beneficial effect during ASIT for pollen allergy, improving clinical symptoms and inducing protective allergen-specific IgG antibodies in humans (19). The most active form of vitamin D_3_, calcitriol (1,25-dihydroxyvitamin D_3_), has also been proposed as adjuvant for SLIT. It was shown to stimulate the production of the anti-inflammatory cytokine IL-10 in human and mouse dendritic cells (20, 21) and to induce intracellular FoxP3 expression in T lymphocytes, a transcription factor expressed by tolerance-promoting regulatory T cells (22). During SLIT, adjuvants are directly applied onto the sublingual epithelium; still, the effect of TLR ligands and calcitriol on the activity of sublingual epithelial cells has not been investigated.

To evaluate whether extracts of house dust mites can modulate immune responses in canine primary sublingual epithelial cells and to evaluate the effect of potential adjuvants during SLIT, sublingual epithelial cells were isolated from biopsies, cultured, and characterized, and the effect of allergens, TLR ligands and calcitriol on the viability, cytokine production, and gene expression by these cells was assessed.

## Materials and Methods

### Isolation of Canine Primary Sublingual Epithelial Cells and Sublingual Subepithelial Cells

Sublingual biopsies of ~0.5 × 0.5 cm were sampled within 3 h after euthanasia from six privately owned dogs that were euthanized for medical reasons unrelated to the study (Supplementary Table A). Oral consent to use the corpse for scientific purposes was given by all dog owners. Of one dog, one-half of the sublingual biopsy was processed for immunohistochemistry. This sample was submerged in Methocel® MC (Merck, Burlington, MA, USA), snap-frozen in liquid nitrogen, and stored at −80°C. To isolate sublingual cells, biopsies were briefly submerged in 70% ethanol, washed three times with Ca^2+^-free and Mg^2+^-free Dulbecco phosphate-buffered saline (DPBS; Thermo Fisher Scientific, Waltham, MA, USA) and incubated in 4 mg/mL Dispase II (Thermo Fisher Scientific) in DPBS on ice. After 16- to 24-h incubation, the epithelial layer was carefully separated from the underlying subepithelial tissue, followed by incubation of the epithelial and subepithelial tissue in DPBS with 0.25% trypsin (Thermo Fisher Scientific) and 2.65 mM EDTA for 20 min at room temperature (RT; 18°C−22°C). Subsequently, cell suspensions were resuspended, trypsin was neutralized with an equal volume of 5% fetal calf serum (FCS) (Merck) in DPBS, and the cells were filtered over a 70-μm cell strainer (Merck). After centrifugation (400 *g*, 10 min, 18°C), cells were counted and 2.5 × 10^5^ cells/cm^2^ were seeded in a culture flask in sublingual epithelial cell culture medium [SECCM; 34 Dulbecco modified eagle medium (DMEM), 14 nutrient mixture F12-Ham [Thermo Fisher Scientific], 5% FCS, 2 nM 3,3′,5-triiodo-l-thyronine sodium salt, 5 μg/mL recombinant human insulin, 10 ng/mL recombinant human epidermal growth factor, 0.4 μg/mL hydrocortisone, 100 nM l-isoproterenol hydrochloride [Merck], 100 U/mL penicillin, 100 μg/mL streptomycin, and 100 μg/mL gentamicin [Thermo Fisher Scientific]) (23, 24). During the first 7 days of culture, the SECCM was supplemented with 2.5 μg/mL amphotericin B (Thermo Fisher Scientific), and medium was changed daily. Thereafter, medium was replaced every 2 to 3 days. Cells were expanded, split (0.25% trypsin, 1 mM EDTA in DPBS) at 70 to 90% confluence, and used at passages 2 to 7 for the experiments. The sublingual epithelial cells were shown to be free of *Mycoplasma* infection during routine testing using the LookOut® Mycoplasma PCR Detection Kit (Merck) according to the manufacturer's instructions.

### Immunofluorescence Staining of Sublingual Tissue

Cryosections (10 μm) of the frozen tissue (LEICA CM3050 S Microtome; Leica, Wetzlar, Germany) on APES-coated glass slides were fixed in acetone for 10 min at −20°C and stained to assess the expression of the integrin subunit α6 (CD49f). In subsequent steps, slides were incubated with PBS + 1% bovine serum albumin (BSA) + 5% goat serum (Merck) at RT for 30 min, with 10 μg/mL rat anti–human CD49f (NKI-GoH3, Thermo Fisher Scientific) or the rat IgG2a anti-KLH control (isotype control; Biolegend, San Diego, CA, USA) for 1 h and with fluorescein isothiocyanate (FITC)–conjugated goat anti–rat IgG (Merck) for 1 h. In between each step, slides were washed with PBS supplemented with 1% BSA. Counterstaining of the nuclei occurred with Hoechst (Thermo Fisher Scientific). Slides were analyzed using a Leica Leitz DMR fluorescence microscope (Leica). Images were processed using ImageJ (25).

### Flow Cytometry

Sublingual epithelial or subepithelial cells (5.0 × 10^4^) were added to conical bottomed wells of a 96-well plate. Ten μg/mL rat anti–human CD49f or isotype control in PBS + 1% BSA was added in a volume of 50 μL for 20 min on ice. Washing of the cells was followed by addition of 50 μL of 0.6 μg/mL Alexa Fluor® 647–conjugated goat anti–rat IgG (Biolegend) in PBS + 1% BSA for 20 min on ice. After two washing steps, cells were resuspended in 100 μL DPBS and analyzed by flow cytometry (CytoFLEX; Beckman Coulter, Brea, CA, USA) using the CytExpert 2.0 software (Beckman Coulter). Singlets were gated in a forward scatter area and height plot, followed by discrimination of cell debris in a forward- and side-scatter plot. Expression of CD49f was assessed by measuring the relative fluorescence intensity for Alexa Fluor® 647.

### Stimulation of Canine Primary Sublingual Epithelial Cells With House Dust Mite Extracts, TLR Ligands, and Calcitriol

Canine primary sublingual epithelial cells were seeded in SECCM in a 24-well plate at 30,000 cells/well and incubated overnight at 37°C and 5% CO_2_. Thereafter, a *Dermatophagoides farinae* extract, a *Dermatophagoides pteronyssinus* extract, TLR ligands, calcitriol, or control medium was added for 24 h to the cultured cells; the supernatant was collected and stored at −20°C. The products, listed in Table 1, were added in a volume of 400 μL serum-free sublingual epithelial cell stimulation medium (SECSM) (34 DMEM, 14 Ham's F-12, 100 U/mL penicillin, 100 μg/mL streptomycin, and 100 μg/mL gentamicin). The *D. farinae* and *D. pteronyssinus* extracts were prepared by the manufacturer (Greer Laboratories, Lenoir, NC, USA) by crushing whole mite bodies followed by a bilevel extraction at 1:10 and 1:5 wt/vol in 0.125 M ammonium bicarbonate and dialysis against distilled water. Because calcitriol was dissolved in ethanol, the control wells for calcitriol contained an equal volume of this solvent. To analyze the effect of the *D. farinae* extract in combination with calcitriol, cells were incubated without stimulus, with 20 μg/mL *D. farinae* extract, or with both *D. farinae* extract and 0.1 μM calcitriol in SECSM. Additionally, after 6-h incubation with 0.1 μM calcitriol and 20 μg/mL *D. farinae* extract, sublingual epithelial cells were lysed in RLT buffer (RNeasy mini kit; Qiagen, Hilden, Germany) and stored at −80°C.

**Table 1:** Products added to the canine primary sublingual epithelial cells.

**Group of products**	**Name (specific receptor)**	**Working concentration**	**Supplier**
Allergen extract	*Dermatophagoides farina* (XPB81D3A2.5, lot: 307244)	20 μg dry protein weight (bradford assay)/mL (2.1 μg/mL Der f 1)	Greer laboratories, Lenoir, NC, USA
	*Dermatophagoides pteronyssinus* (XPB82D3A2.5, lot: 346230)	20 μg dry protein weight (bradford assay)/mL (0.58 μg/mL Der p 1)	
TLR ligands	FSL-1 (TLR2)	1 μg/mL	Invivogen, San Diego, CA, USA
	Heat-killed *Listeria monocytogenes* (HKLM) (TLR2)	10^8^ cells/mL	
	Pam3CSK4 (TLR2)	1 μg/mL	
	Polyinosinic:polycytidylic acid high molecular weight (poly I:C HMW) (TLR3)	10 μg/mL	
	Polyinosinic:polycytidylic acid low molecular weight (poly I:C LMW) (TLR3)	10 μg/mL	
	LPS (*Escherichia coli* K12) (TLR4)	10 μg/mL	
Vitamin D	Calcitriol (vitamin D receptor)	1–0.01 μM	Merck, Burlington, MA, USA

### MTT Assay

An MTT assay was used to evaluate the effect of the different stimuli on the viability and metabolic activity of canine primary sublingual epithelial cells. Five thousand cells/well in 200 μL SECCM were seeded in wells of a 96-well cell culture plate. After overnight incubation at 37°C and 5% CO_2_, medium was replaced by the products in Table 1 in SECSM. Following an additional 20-h incubation at 37°C and 5% CO_2_, plates were developed as described by Kieckens et al. (26), with some minor adjustments. Briefly, 20 μL of 5 mg/mL MTT [3-(4,5-dimethyl-2-thiazolyl)-2,5-diphenyl-2H-tetrazolium bromide] in Hanks balanced salt solution (Thermo Fisher Scientific) was added to each well, followed by 4-h incubation (37°C, 5% CO_2_). After removal of the supernatant, the formed formazan crystals were dissolved in 1:1 (vol/vol) ethanol/dimethyl sulfoxide using gentle resuspension, followed by measurement of the optical density (OD) at 560 nm (OD1) and 620 nm (OD2). OD2 was subtracted from OD1, and results were finally expressed as the percentage viable cells by dividing OD values of the stimulated wells by those of the unstimulated control.

### Cytokine Enzyme-Linked Immunosorbent Assays

The secretion of the proinflammatory cytokine IL-6, the chemokine IL-8/CXCL8, and the anti-inflammatory cytokine IL-10 in cell-free supernatants was measured using canine-specific DuoSet enzyme-linked immunosorbent assay (ELISA) kits (R&D Systems, Minneapolis, MN, USA) according to the manufacturer's instructions. Human and canine transforming growth factor β1 (TGF-β1) were shown to have high homology (27), and therefore, the human TGF-β1 ELISA was used to detect canine TGF-β1 (28).

### RNA Extraction and RNA-Sequencing

RNA extraction and DNase treatment of the samples was performed using the RNeasy mini kit and Rnase-Free DNase Set (Qiagen) according to the manufacturer's instructions. Extracted RNA was stored at −80°C. Total RNA concentration was measured using the Quant-iT RiboGreen RNA Assay Kit (Thermo Fisher Scientific). RNA quality was inspected using an RNA 6000 Nano chip on a BioAnalyser (Agilent Technologies, Santa Clara, CA, USA). For each sample, a sequencing library was constructed using the QuantSeq 3′ mRNA-Seq Library Prep Kit FWD for Illumina (Lexogen, Vienna, Austria) starting from 500 ng total RNA and amplified by 14 polymerase chain reaction (PCR) cycles. Quality and size distribution of the libraries were checked on a BioAnalyser (Agilent Technologies) using the high-sensitivity Quant-iT dsDNA Assay Kit (Thermo Fisher Scientific) and a high-sensitivity DNA chip. Library concentration was determined according to Illumina's Sequencing Library qPCR Quantification Guide on a LightCycler 480 (Roche Life Science, Penzberg, Germany). Sequencing of the libraries was done at Ghent University's NxtGnt sequencing facility as single-read 75 bp on a NextSeq 500 sequencer (Illumina, San Diego, CA, USA) using four lanes per sample. Subsequently, sequencing read quantity and quality were checked using FastQC (v0.11.8). Contamination was determined using FastQ Screen (v0.14.0). Adapter trimming and removal of reads having a quality score lower than 20 or containing any ambiguities were done using cutadapt (v2.5). The remaining high-quality reads were mapped on the CanFam3.1 reference dog genome using STAR (v2.7.2), and features were count at the gene level using RSEM (v1.3.1).

### Statistical Analysis

Statistical analysis was performed using R [R Core Team (29)]. Normal distribution of the data was assessed using the Shapiro–Wilk test and quantile–quantile plot. Log-transformed ELISA concentrations and untransformed data of the MTT test were normally distributed and analyzed using the paired Student *t*-test. A *p* < 0.05 was considered statistically significant. Differential expression analyses of the RNA-sequencing data were done in R using the edgeR (v3.26.8) package. For each analysis, feature counts were normalized using edgeR's standard normalization method. To remove uninformative low-count features, those having <1 cpm (counts per million) in the number of samples equivalent to the size of the smallest group were discarded. Statistical testing was done by fitting a general linear model, followed by a quasi-likelihood F test. Differentially expressed features were considered statistically significant when having a fold change of at least 2 (doubling or halving between the compared groups) and a false discovery rate (or adjusted *p*-value according to Benjamini–Hochberg) smaller than or equal to 0.05.

## Results

### A *D. farinae* Extract and TLR Ligands Increase CXCL8 Secretion While Calcitriol Suppresses It

**Figure 1:**
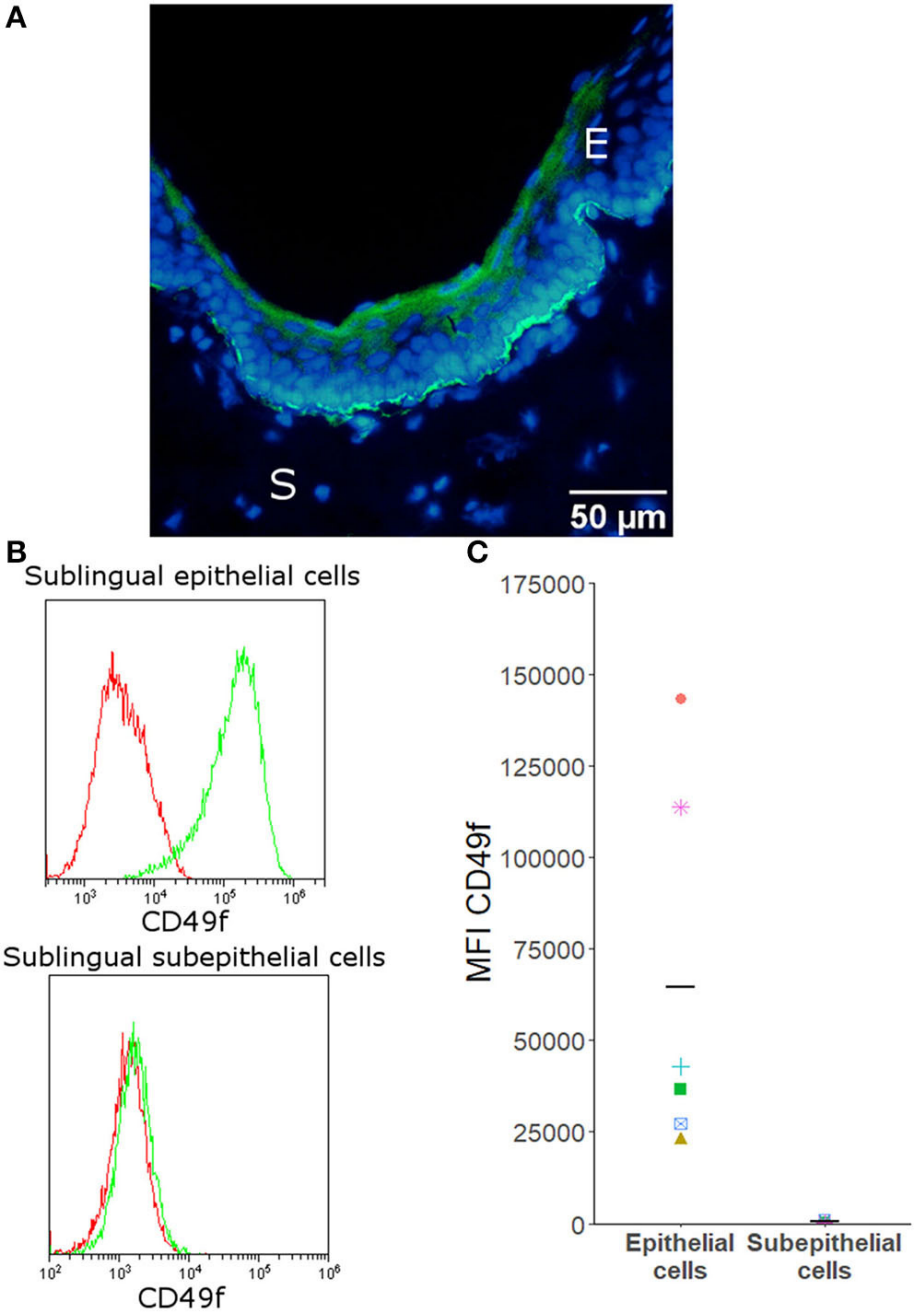
CD49f is expressed by canine sublingual epithelial, but not by sublingual subepithelial cells. **(A)** Immunofluorescence of canine sublingual tissue in which cryosections were stained with CD49f (FITC) and counterstained with Hoechst. E = epithelium, S = subepithelial tissue. **(B)** Histograms generated by flow cytometric analysis that are representative for all donors. Isotype controls are given in red; stained cells are shown in green. **(C)** Flow cytometric analysis: mean fluorescence intensity (MFI) of all samples stained with CD49f for sublingual epithelial cells (*n* = 6) and subepithelial cells (*n* = 4). Mean fluorescence intensity of the isotype control was subtracted from the MFI of the stained cells. Horizontal bars indicate the mean.

To assess whether two house dust mite extracts might modulate cytokine secretion by canine primary sublingual epithelial cells, cells were isolated from sublingual biopsies. Immunofluorescence staining and flow cytometry showed that the isolated sublingual cells stained positive for the epithelial marker CD49f (Figure 1).

**Figure 2:**
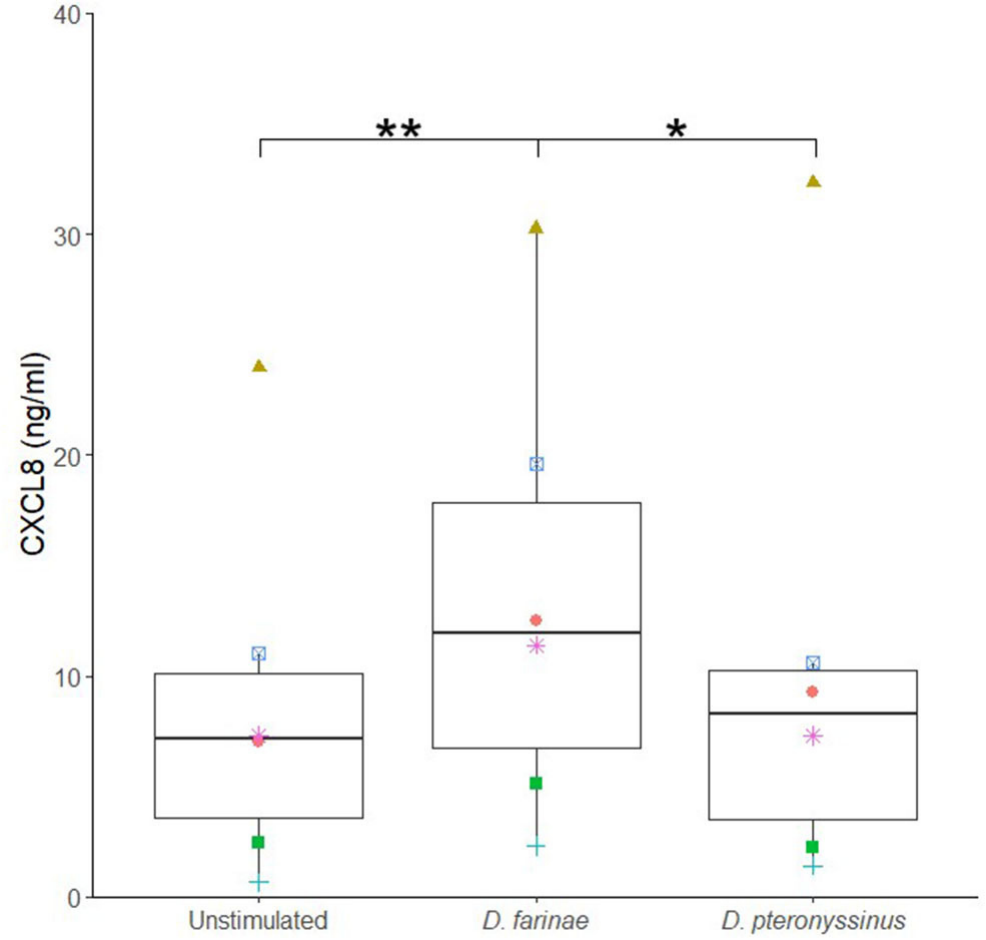
A *Dermatophagoides farinae* extract significantly increases CXCL8 secretion by canine sublingual epithelial cells (*n* = 6). The cells were cultured for 24 h with 20 μg/mL *D. farinae* extract or 20 μg/mL *D. pteronyssinus* extract and CXCL8 secretion was measured. *p < 0.05; **p < 0.01 to control; paired Student *t*-test.

Next, it was evaluated whether an allergen extract of *D. farinae* and *D. pteronyssinus* could alter the sublingual epithelial cells' cytokine secretion. The *D. farinae* extract induced a significant increase in CXCL8 secretion as compared to the unstimulated controls and *D. pteronyssinus* extract (Figure 2). Therefore, we used this *D. farinae* extract to evaluate the effect of potential adjuvants on the CXCL8 response.

**Figure 3:**
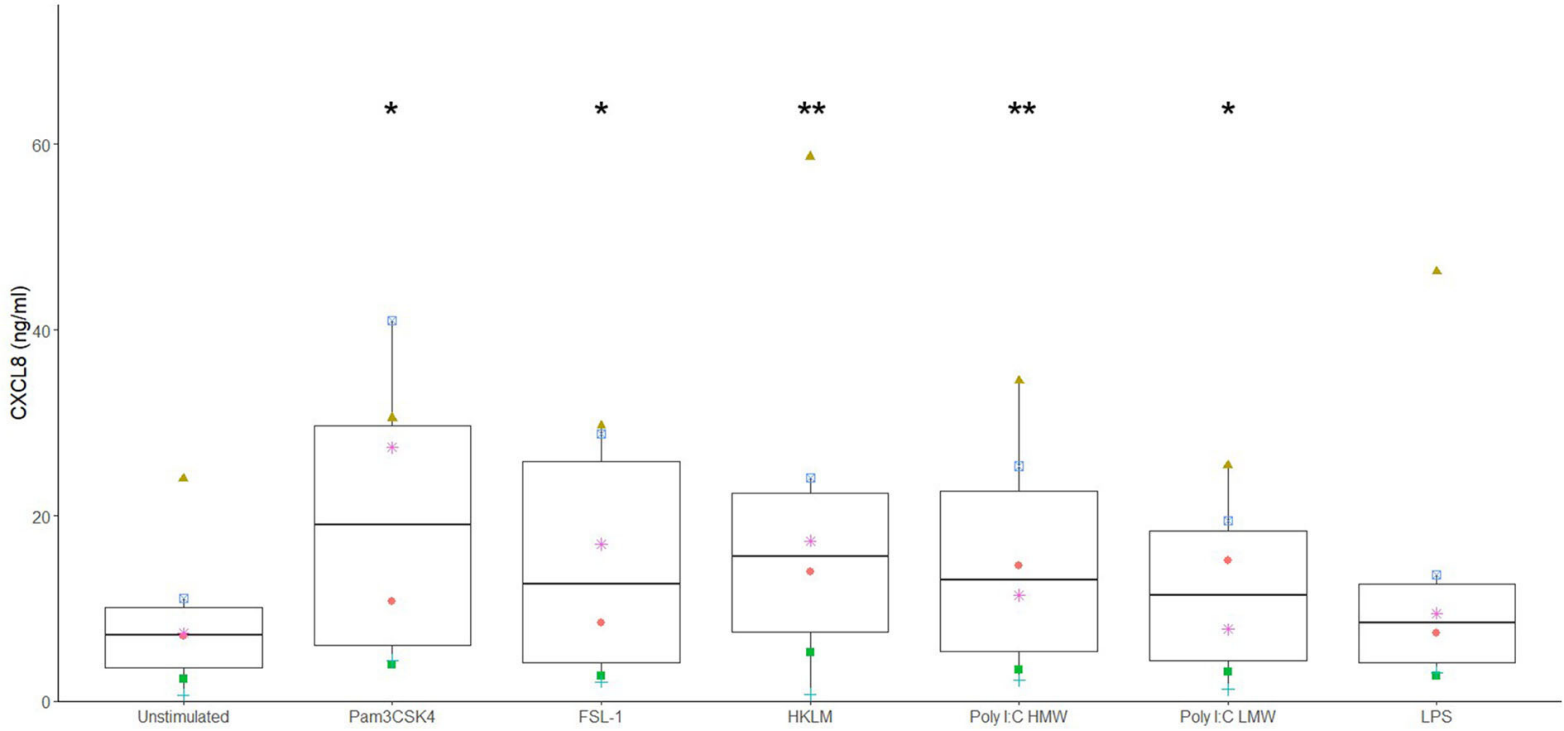
Ligands for TLR2 and TLR3 significantly increase CXCL8 secretion by canine sublingual epithelial cells (*n* = 6). The cells were cultured for 24 h with Pam3CSK4 at 1 μg/mL, FSL-1 at 1 μg/mL, HKLM at 10^8^ cells/mL, poly I:C high (HMW) and low molecular weight (LMW) at 10 μg/mL, and LPS at 10 μg/mL, and CXCL8 secretion was measured by ELISA. *p < 0.05; **p < 0.01 to control; paired Student *t*-test.

**Figure 4:**
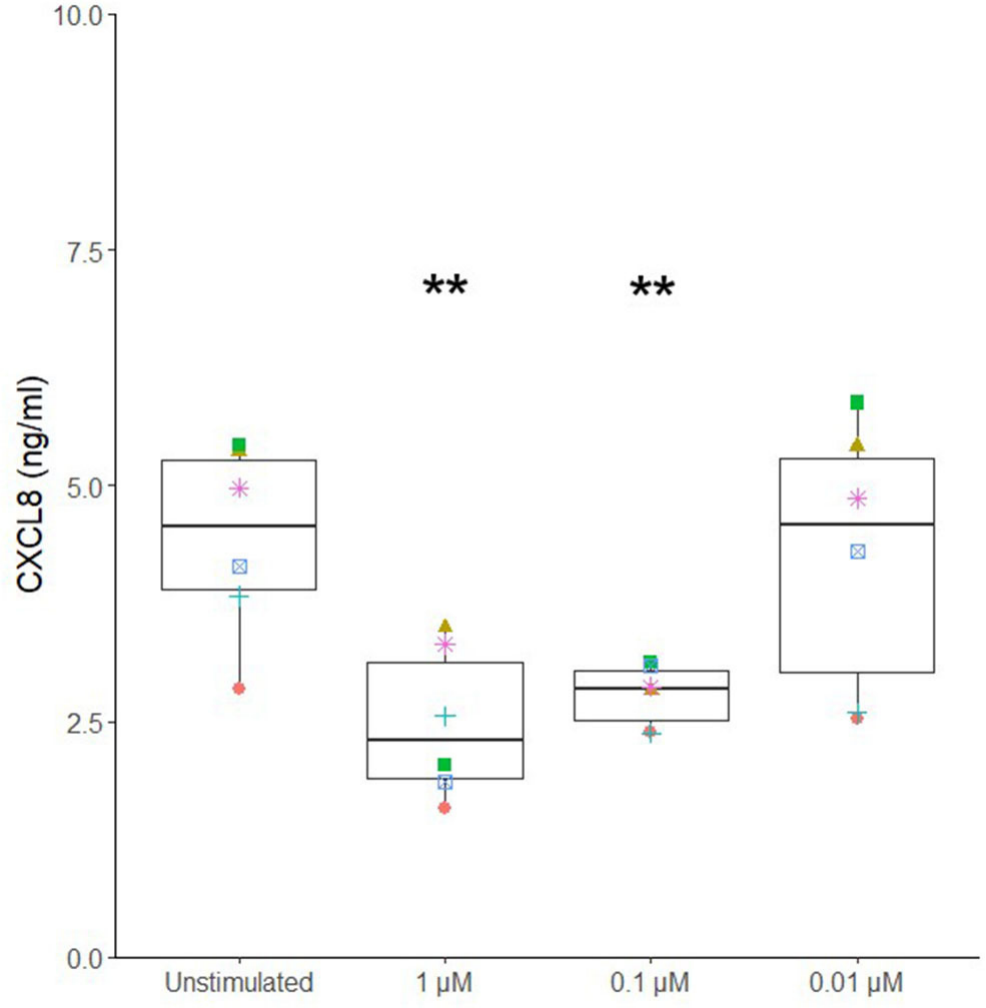
Calcitriol significantly suppresses CXCL8 secretion by canine sublingual epithelial cells (*n* = 6). The cells were stimulated with 1, 0.1, and 0.01 μM calcitriol for 24 h, and CXCL8 secretion was measured by ELISA. **p < 0.01 to control; paired Student *t*-test.

Different potential adjuvants were tested for their direct effect on cytokine secretion by canine sublingual epithelial cells. The TLR2 ligands, FSL-1, HKLM, and Pam3CSK4, and TLR3 ligands, poly I:C high (HMW) and low molecular weight (LMW), significantly increased CXCL8 secretion by sublingual epithelial cells (*p* < 0.05; Figure 3). Calcitriol was able to significantly suppress the CXCL8 secretion at concentrations of 1 and 0.1 μM (*p* < 0.05), but not at 0.01 μM (Figure 4). The sublingual epithelial cells constitutively produced TGF-β1 (unstimulated control: 41 ± 15 pg/mL; median ± interquartile range), and this did not change significantly when incubated with the *D. farinae* extract, TLR ligands, or calcitriol (data not shown). Neither unstimulated nor stimulated sublingual epithelial cells detectably produced the proinflammatory cytokine IL-6 or the anti-inflammatory cytokine IL-10.

**Figure 5:**
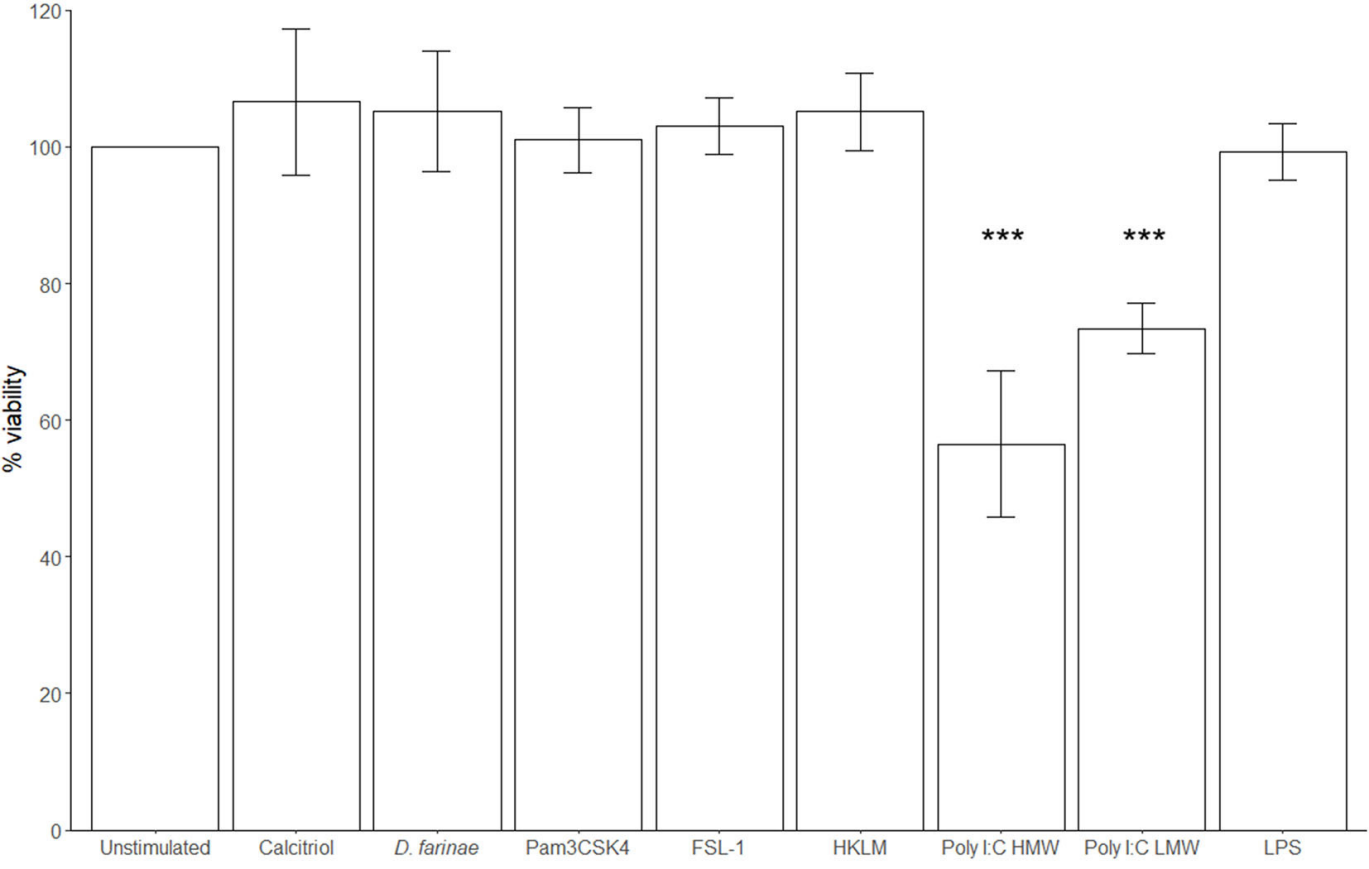
The TLR3 ligand poly I:C significantly decreases the viability of canine sublingual epithelial cells (*n* = 6). The cells were cultured for 24 h with 0.1 μM calcitriol, *D. farinae* extract at 20 μg/mL, Pam3CSK4 at 1 μg/mL, FSL-1 at 1 μg/mL, HKLM at 10^8^ cells/mL, poly I:C high (HMW) and low molecular weight (LMW) at 10 μg/mL, and LPS at 10 μg/mL, and the effect on cell viability was assessed using an MTT test. ***p < 0.001 to control; paired Student *t*-test. Error bars: standard deviation.

Neither *D. farinae* extract at 20 μg/mL, nor the TLR ligands FSL-1 at 1 μg/mL, HKLM at 10^8^ cells/mL, Pam3CSK4 at 1 μg/mL, lipopolysaccharide (LPS) at 10 μg/mL, or calcitriol at 0.1 μM had a significant effect on cell viability after 24-h incubation. Only poly I:C HMW (57% ± 12%; mean ± standard deviation) and LMW (73% ± 5%) significantly decreased the viability of canine sublingual epithelial cells (*p* < 0.001) (Figure 5).

### Calcitriol Can Suppress CXCL8 Secretion by Canine Sublingual Epithelial Cells Even in the Presence of *D. farinae*

**Figure 6:**
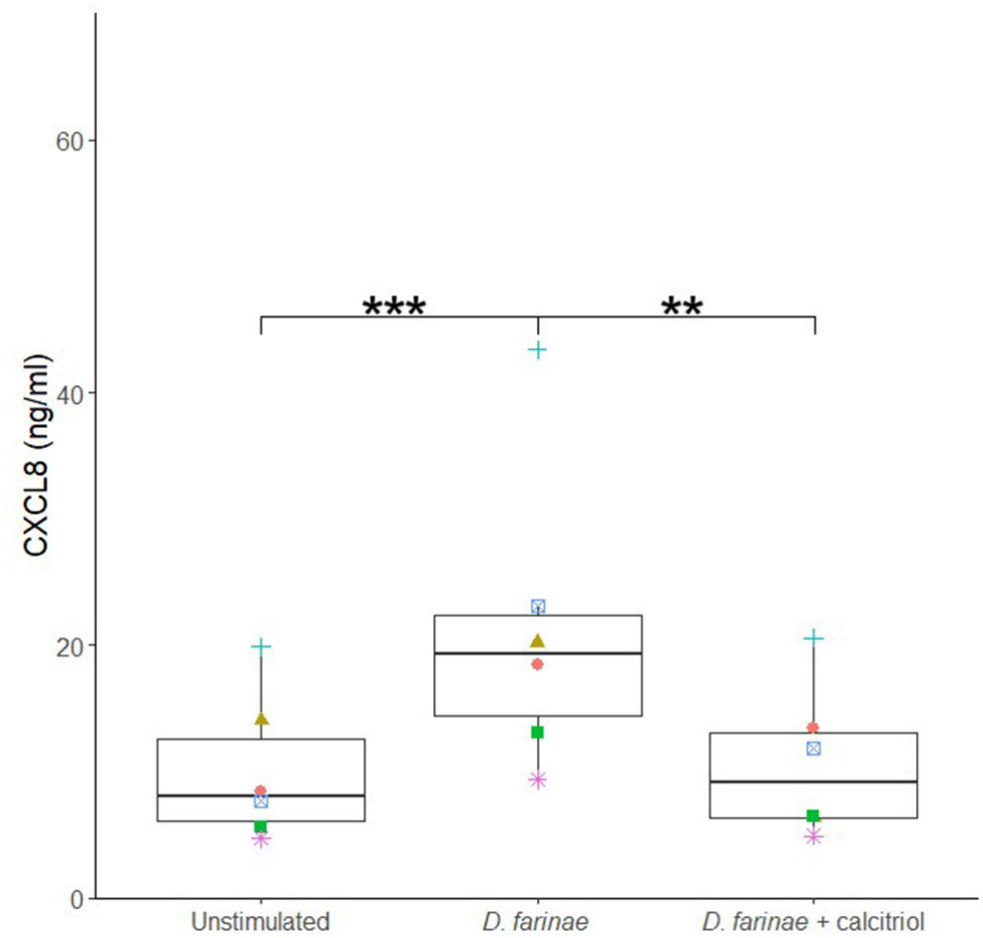
Coadministration of a *D. farinae* extract with calcitriol suppresses CXCL8 secretion by canine sublingual epithelial cells (*n* = 6). The cells were cultured with 20 μg/mL *D. farinae* extract or a combination of 20 μg/mL *D. farinae* extract with 0.1 μM calcitriol for 24 h, and CXCL8 secretion was measured by ELISA. **p < 0.01; ***p < 0.001 to control; paired Student *t*-test.

To investigate the effects of calcitriol on canine sublingual epithelial cells when coadministered with the extract of *D. farinae*, cells were incubated with *D. farinae* extract alone, a combination of *D. farinae* extract with calcitriol, or with medium without stimulus. Costimulation with calcitriol and *D. farinae* significantly suppressed CXCL8 production as compared to cells stimulated with *D. farinae* alone (*p* < 0.05). There was no significant difference in CXCL8 secretion between cells stimulated with *D. farinae* and calcitriol combined and cells cultured without any stimulus, suggesting maintenance of normal CXCL8 secretion levels (Figure 6).

### Calcitriol Induces Glucocorticoid Receptor and Prostaglandin E Synthase mRNA Expression

No significantly differentially expressed genes could be identified when comparing RNA-sequencing data of sublingual epithelial cells incubated with and without *D. farinae* extract. Calcitriol, however, did significantly upregulate 13 genes compared to unstimulated cells, among which NR3C1, the glucocorticoid receptor; CYP24A1, a cytochrome P450 that catabolizes vitamin D; and PTGES, an enzyme that generates prostaglandin E (Table 2). Interestingly, in all the conditions tested, transcript counts of less than 1 count per million were observed for IL-1α, IL-1β, IL-6, IL-10, IL-25, IL-33, and TSLP.

**Table 2:** Calcitriol-induced gene expression in canine sublingual epithelial cells (*n* = 5). Cells were incubated with 0.1 μM calcitriol for 6 h.

**Gene**	**Fold change**	**Adjusted *p*-value**
NR3C1	2.21	0.004
CYP24A1	500	0.008
C28H10orf90	5.65	0.008
PTGES	1.95	0.012
KALRN	1.59	0.013
HMOX1	3.82	0.022
OSGIN1	22.22	0.022
SLC4A7	2.23	0.027
SEMA4D	3.24	0.029
KIF26A	1.95	0.037
KCNK10	14.08	0.038
LMCD1	2.26	0.044
TMC7	1.94	0.045

*Fold changes to the unstimulated control are given*.

## Discussion

In this study, primary sublingual epithelial cells from dogs were isolated from biopsies, cultured *in vitro*, and characterized for expression of the marker CD49f. All sublingual epithelial cells stained positive for this marker. This is in agreement with findings in humans and dogs, which showed that CD49f is a reliable marker to identify epithelial cells (30–33).

Canine sublingual epithelial cells were stimulated with a *D. farinae* extract, a *D. pteronyssinus* extract, different TLR ligands, and calcitriol to assess differences in their cytokine and chemokine secretion profile. Only the production of CXCL8 could be significantly modulated. This proinflammatory chemokine is capable of attracting neutrophils and T lymphocytes (34, 35). The oral cavity contains low numbers of proinflammatory effector cells (36, 37), and the absence of proinflammatory signals likely explains the facilitated generation of antigen-specific tolerance. During SLIT, allergens are administered onto sublingual epithelial cells. These cells showed a significant increase in CXCL8 secretion when stimulated with a *D. farinae* extract. Also, in human and canine epidermal keratinocytes, crude extracts of *D. farinae* and its allergenic protein, Der f 1, increased the expression of proallergenic mediators and proinflammatory cytokines (11, 38). It should be noted that, in both cited studies, the allergen exposure protocol was insufficiently documented, and that in the study of Maeda et al. (38), the activation state and purity of the Der f 1 were not assessed. It should also be mentioned that, in order to generate more representative results for the sublingual epithelial cells, a mimic of the mucosal lining fluid and saliva could have been used rather than serum-free medium. While another study showed that *D. pteronyssinus* proteins can induce proinflammatory cytokine expression in epithelial cells (8), the *D. pteronyssinus* extract we used did not modulate cytokine secretion by the sublingual epithelial cells. Differences in composition of the mite extracts could explain this discrepancy in response (39). Nonetheless, the *D. farinae* extract did induce CXCL8 secretion by canine sublingual epithelial cells, which indicates that application of such an extract onto the sublingual epithelium has the potential to generate a proinflammatory response in the oral mucosa.

When the effect of different TLR ligands was investigated, the TLR2 ligands Pam3CSK4, HKLM, and FSL-1, and the TLR3 ligand, poly I:C, but not LPS, significantly stimulated CXCL8 secretion. In the past, TLR2 ligands were shown to be promising adjuvants for allergy treatment in some studies (16–18), whereas other studies with mouse asthma models showed that TLR2 ligands could enhance proallergenic responses (40, 41). In canine epidermal keratinocytes, LPS and Pam3CSK4 also promoted the secretion of proinflammatory and proallergenic mediators (42–44). Additionally, the TLR3 ligand, poly I:C, had a significant lethal effect on the sublingual epithelial cells, as was observed for bronchial epithelial cells as well as in epidermal keratinocytes (45, 46). The use of TLR ligands during SLIT is therefore likely to have a negative impact on the tolerance-promoting anti-inflammatory environment of the oral mucosa.

Calcitriol or 1,25-dihydroxyvitamin D_3_ is a small lipophilic molecule that easily penetrates cell membranes through diffusion (22, 47), after which it can bind to the vitamin D receptor of epithelial cells and regulate several genes (48). Interestingly, in combination with the *D. farinae* extract, calcitriol significantly reduced CXCL8 secretion by canine sublingual epithelial cells. During SLIT, calcitriol therefore seems promising to prevent an allergen extract–induced increase in CXCL8 secretion by sublingual epithelial cells. Interestingly, calcitriol significantly upregulated the mRNA expression of NR3C1, the receptor for glucocorticoids. Indeed, calcitriol and the glucocorticoid dexamethasone were shown to have a combined tolerance-promoting effect when sublingually administered in a mouse model (21). Additionally, augmented expression of the prostaglandin E–generating enzyme PTGES was observed. Prostaglandin E has both proinflammatory and anti-inflammatory properties, being capable of activating mast cells (49), whereas secretion by bronchial epithelial cells has an anti-inflammatory effect on murine dendritic cells (50). Calcitriol can have an ambiguous effect on the proliferation of human epithelial cells, with low concentrations (10^−9^–10^−11^ M) promoting the proliferation of human epidermal keratinocytes, whereas higher concentrations (>10^−8^ M) inhibited keratinocyte proliferation *in vitro* (51, 52). However, in our hands, calcitriol did not affect the viability and metabolic activity of canine sublingual epithelial cells, as was shown for human bronchial epithelial cells (53). Nevertheless, if calcitriol would be used as an adjuvant during SLIT, the effects of repeated administration on the oral mucosa should be investigated.

Although low concentrations of TGF-β1 could be detected in canine sublingual epithelial cell supernatant, neither calcitriol nor the TLR ligands or the *D. farinae* extract significantly changed the secretion of this cytokine. In humans, calcitriol was shown to induce TGF-β in epidermal keratinocytes (48), whereas incubation of primary oral epithelial cells with bacteria suppressed the secretion of this cytokine (54). In the context of allergies, TGF-β generally leads to allergen-tolerating responses by inducing regulatory T cells and promoting an isotype shift in B cells in favor of IgA production (55, 56). Substances that increase TGF-β secretion by sublingual epithelial cells would therefore be interesting candidates for use as adjuvants during SLIT. While production of IL-6 and IL-10 has been observed in other types of epithelial cells (57–63), no secretion of these cytokines was detected for canine sublingual epithelial cells by ELISA or RNA sequencing. Furthermore, proallergenic mediators associated with the epithelium, TSLP, IL-25, and IL-33 (4, 64) and the proinflammatory cytokines IL-1α and IL-1β were not expressed by canine sublingual epithelial cells. Whether this difference in mediator expression compared to epithelial cells of other tissues is due to specific properties of the canine sublingual epithelium should be investigated.

In this study, canine sublingual epithelial cells were cultured as a monolayer. However, the sublingual epithelium consists of 12–18 layers in dogs (65). This difference might limit the conclusions that can be drawn for *in vivo* applications. Indeed, cytokine secretion repertoires of epidermal keratinocytes differed when cultured as a monolayer compared to a more complex multilayered culture model (39). Nevertheless, unlike the skin, the canine sublingual epithelium is non-keratinized tissue, which allows penetration of molecules to the level of the basal epithelial cells to exert their effects (37, 65). This study therefore provides relevant insights into which mediators can be produced by sublingual epithelial cells positioned close to the basement membrane, mediators that could influence the behavior of local immune cells.

In conclusion, canine primary sublingual epithelial cells were isolated from dogs and showed increased secretion of CXCL8 in response to a *D. farinae* extract, TLR2, and TLR3 ligands, whereas the production of this chemokine could be suppressed by calcitriol. Furthermore, when applied onto the cells together with an extract of *D. farinae*, calcitriol downregulated CXCL8 concentrations to levels similar to those observed in unstimulated sublingual epithelial cells. As such, calcitriol seems to be a promising adjuvant for use during SLIT because it might help to maintain the tolerance-inducing properties of the oral mucosa during desensitizing treatments.

## Data Availability Statement

All datasets generated for this study are included in the article/Supplementary Material. The RNA-sequencing data discussed in this publication have been deposited in NCBI's Gene Expression Omnibus (66) and are accessible through GEO Series accession number GSE148592 (https://www.ncbi.nlm.nih.gov/geo/query/acc.cgi?acc=GSE148592).

## Ethics Statement

Ethical review and approval was not required for the animal study because the samples were collected from corpses of animals that were euthanised for reasons independent of this study. Oral informed consent was given by all owners.

## Author Contributions

EC conceptualized the study. MP, CH, and CW performed the experiments, acquired, and analyzed the data. MP drafted the manuscript. BD, HD, and EC gave valuable input on the revision of the manuscript. FV contributed to the design of the RNA-sequencing experiment. YG and FV performed the statistical analysis on the RNA-sequencing data. All authors contributed to manuscript revision, read, and approved the submitted version.

## Conflict of Interest

The authors declare that the research was conducted in the absence of any commercial or financial relationships that could be construed as a potential conflict of interest.
