# Supplementary material for: Adjuvanting Allergen Extracts for Sublingual Immunotherapy: Calcitriol Downregulates CXCL8 Production in Primary Sublingual Epithelial Cells
Source: Front Immunol. 2020 Jun 9;11:1033. doi: 10.3389/fimmu.2020.01033 (PMC7295906; doi:10.3389/fimmu.2020.01033)
Supplement: Supplementary file 1 [file Table_1.DOCX]

**Supplementary Table A**

| **Breed** | **Sex** | **Age (years)** | **Reason for euthanasia** |
| --- | --- | --- | --- |
| American Staffordshire | Male intact | 13 | Neurological complaints |
| Bernese mountain dog | Male neutered | 4 | Persistent anemia |
| Labrador retriever | Male intact | 13 | Geriatric complaints |
| Mixed-breed | Male intact | 11 | Hemangiosarcoma |
| Newfoundlander | Male intact | 7 | Heart disease |
| Rottweiler | Female intact | 3 months | Severe gastro-intestinal complaints |
